# Hospital and Institutional News

**Published:** 1917-02-24

**Authors:** 


### HOSPITAL AND INSTITUTIONAL NEWS.

### SIR COOPER PERRY'S ACCIDENT.

On Friday of last week, owing to the weather, Sir Cooper Perry met with an accident which at first caused him grievous pain, and has confined him to bed. When a diagnosis could be made the dislocation was successfully reduced. We are glad to report that Wednesday morning's report was both encouraging and satisfactory. Sir Cooper has become such a tower of strength in some of the larger organisations which affect the best interests of the hospitals and those connected with them, that his speedy recovery will be welcomed by all. This is now hoped for, to the relief of many who have had the pleasure of working with him and knowing the supreme value of his many services, precise knowledge, and great acumen. The sympathy of his many friends will cheer the patient and be grateful to Lady Perry too.

### OPHTHALMIA NEONATORUM.

The discussion of measures for diminishing the ravages of this disease, which took place the other day at the meeting of the Central Council for District Nursing, prompted the medical correspondent of the *Times* to a depreciation of the work of the voluntary hospitals in dealing with this disease. It is easy enough to agree that this disease is preventable and should therefore be prevented, and that when it occurs sight can nearly always be saved by prompt treatment; but this is entirely a different matter from the inference that "all these cases should be treated in hospitals controlled by the State." Nor is it true, in our opinion, that "this is a serious matter which has been allowed to drift to a great extent." It may be that some voluntary hospitals are not as alive as they should be to the importance of very active and efficient measures in dealing with ophthalmia neonatorum; but, if so, it is true of a very small minority indeed. Also, the last ten years has witnessed a great growth of purely voluntary effort, mainly at the instigation of the staffs of voluntary hospitals, to extirpate this disease; and it is nothing short of a libel on the hospitals of this country to state that the matter has been allowed to drift. Further, it must not be forgotten that this disease is usually, though not always, of venereal origin, and that some hospitals are still precluded by their constitution from accepting such cases. Presumably, the venereal clinics now being established all over the country will be expected to treat ophthalmia neonatorum and other forms of *lues insontium*. The proper remedy for any shortage of accommodation that there may be for cases of this sort is that indicated in the report presented to the Central Council of District Nursing—namely, the recommendation "that representation be made to King Edward's Hospital Fund for London indicating the need that exists for greater facilities for in-patient treatment of mother and child"; and, it may be added, to abide by the decision arrived at by that body.

### THE WORK OF A GREAT MANCHESTER HOSPITAL.

Some interesting facts are contained in the annual report of the Manchester Royal Infirmary. The board of management remarks that the anxieties of carrying on the work efficiently in the third year of the war were aggravated by the further depletion of the staff owing to military calls. At the urgent request of the War Office 116 additional beds were set apart for the treatment of wounded soldiers, bringing the total accommodation for military patients up to 424. During the twelve months 2,411 soldiers were admitted. Notwithstanding the further increase of accommodation for wounded soldiers, the waiting list of civilian patients was considerably reduced. The work of the Cancer Research Laboratory has been temporarily suspended in consequence of the medical officer in charge being now engaged in military duties. The board emphasises the need for continued financial support, and points out that £8,500 for the Royal Infirmary and £3,500 to meet the annual deficit on the Barnes Convalescent Hospital at Cheadle are urgently required. The total number of patients treated during the year was 59,566.

### A LATE DIRECTOR-GENERAL OF THE INDIAN MEDICAL SERVICE.

The death is announced of Surgeon-General Sir Benjamin Franklin, K.C.I.E., at the age of seventy-two. This distinguished officer was formerly Director-General of the Indian Medical Service and Sanitary Commissioner with the Government of India. He was surgeon to Lord Elgin, when Viceroy of India, from 1894 to 1898; he afterwards became honorary physician to Queen Victoria, King Edward, and King George. He was also British delegate to the International Sanitary Conference in Rome in 1907 and at Paris in 1911-12. Until quite recently he had been actively engaged in work for the Red Cross Society, especially in connection with the Roehampton Hospital, near which he lived. He was a Knight of the Order of St. John of Jerusalem in England, and took a great interest in the work of the Ambulance Association of that Order.

### DEATH OF A WELL-KNOWN OPHTHALMIC SURGEON.

By the death of Mr. W. H. H. Jessop St. Bartholomew's Hospital loses the senior ophthalmic surgeon and the Ophthalmological Society its President. Mr. Jessop, who was in his sixty-fourth year, was very well known to the whole medical profession by reason of his elementary textbook on eye diseases, which has for some years been one of the most popular books in this specialty amongst students and general practitioners. He was also very popular amongst his ophthalmological colleagues, both at home and abroad; for he was a constant visitor to international congresses and was always on the alert to uphold the dignity and advance the interests of the specialty to which his life-work was devoted. Mr. Jessop was educated at Cambridge and at St. Bartholomew's Hospital; he was a Bachelor of Surgery and of Medicine at his old University, a Fellow of the Royal College of Surgeons of England, and a Justice of the Peace. His death was caused by pneumonia, after a brief illness. A quite recent achievement in which he took a leading share is the amalgamation of the three competing periodicals devoted to ophthalmology into the *British Journal of Ophthalmology*, which Sir Anderson Critchett says "has already started on a most promising career, and will remain a lasting ornament to the tact, energy, and enterprise of its chief founder."

### THE CAT AS A CARRIER OF DISEASE.

Since the publication of the paper on this topic by Mr. Arnold Lawson, and the introductory remarks which were prefixed to it (The Hospital, February 17, p. 405), a distinguished ophthalmologist writes to say that within the last fortnight he has seen a case of infection transferred from a cat to a boy of three years old. The cat had a discharge from the eye, from which the child contracted a very severe conjunctival infection; the same organism was isolated from the cat and from the child. For a day or two it seemed doubtful whether the child's sight could be saved, but, fortunately, the issue was favourable. If any confirmation were needed of the warning we gave a fortnight ago to the public and the medical profession of the danger of allowing children or young people to be too intimate with cats, this case surely supplies it. We commend the whole subject to the very careful attention of our readers, both lay and medical; for we are convinced it is a matter of much greater importance than is commonly realised. It is for the specialists in ophthalmic surgery to educate the rest of the profession, and for them in turn to educate the public.

### WHEN DOCTORS DIFFER.

A most extraordinary case has recently been before the Hull Tribunal under the Military Service Acts, which has resulted in the complete vindication of the Medical Board in that city, and the humiliation of three civil practitioners there. A certain young man desired to evade Army service, and applied to the Tribunal for exemption on various grounds, one of which was medical unfitness. In support of this claim he produced elaborate certificates from three local doctors to the effect that he had severe and long-standing middle-ear disease, in which acute mastoid or cerebral complications might arise at any moment. One doctor even said that operation within a week was his only safeguard. The Medical Board could find no evidence of ear disease whatever, so the matter was referred to a distinguished London specialist, who entirely confirmed the Board's view, and submitted a report explaining fully why he disbelieved altogether the history of years of disease given by the patient and endorsed by his doctors. It is obvious on the face of matters that one or other of two things has occurred in this case. Either the patient has been personated by someone else at his supposed interviews with the certifying doctors —that is, a fraud has been perpetrated. Or else the three Hull medical men have been guilty of abominable carelessness in certifying the presence of definite abnormalities which do not in fact exist, whereby the ends of justice might have been defeated. It should not be omitted that it turned out during the hearing of the case that a fourth civilian doctor who had seen the patient declined to give him any certificate of disability for military service, a fact which he concealed as long as possible from the Tribunal. We certainly think the documents of this case should be submitted to the General Medical Council.

### COAL DIFFICULTIES AT GUY'S.

The report of the consulting engineer of Guy's Hospital for 1916 deals largely with the increase in the price of coal and the precautions taken by the executive to ensure the considerable supply requisite for the work of this great hospital. The average price per ton paid has steadily increased from 16s. 6d. per ton in 1910 to 36s. 5d. in 1916; the amount expended during 1916 was £10,728, as against £6,966 in the previous year. Fortunately, the contractors who had good facilities for supplying sea-borne coal were able to keep the hospital well supplied up to the date of the report, but as the various activities of the hospital could not be allowed to suffer through shortage, a site for storage was arranged in the neighbourhood and a quantity was stacked amounting to nearly 600 tons. The cost of coal naturally affects the cost of supplying electricity for power and light, and the unit has increased in five years from .98 to 1.62 of a penny; the units used, owing to additional requirements, are 20 per cent. higher than they were five years ago, and this fact, of course, should have had a tendency to decrease the cost per unit, and the fact should be taken into consideration in examining the figures when so divided.

### RECOGNITION FOR PHILANTHROPIC WORKERS.

The useful work performed by a number of well-known Leicester municipal and philanthropic workers has been recognised by an addition of several names to the Commission of the Peace for the borough. Mr. T. Fielding Johnson, jun. (J.P. for the county), and Colonel C. J. Bond, F.R.C.S., are respectively chairman and vice-chairman of the board of the Royal Infirmary. Mr. J. Gipson Clarke is a life governor of that institution and a vice-president of the Saturday Hospital Society, of which he is chairman of the Finance Committee. Mr. A. I. Groves is the founder and president of the Leicester Cripples' Guild and a member of the board of the infirmary. Other members of the board of that institution similarly honoured are Alderman Arthur Tollington (chairman of the Finance Committee of the Corporation), Alderman George Chitham (chairman of the Estates Committee), Councillor W. E. Hincks (chairman of the Watch Committee), and Mr. H. H. Woolley, the organising secretary of the Leicester Saturday Hospital Society. The useful knowledge these experienced philanthropic workers have gained cannot fail to be of assistance in the administration of justice. The indirect recognition of an important institution like the Royal Infirmary is another step in the direction of closer unity between voluntary institutions and municipalities, the need for which has recently been emphasised in the scheme of the Local Government Board for the treatment of venereal diseases, and will be further emphasised after the war in co-ordinated plans for the surgical and orthopædic treatment of discharged soldiers, and of school children suffering from physical defects.

### THE BISHOP OF ST. ASAPH ON WAR MEMORIALS.

COMMENTING at the annual meeting of the Royal Alexandra Children's Hospital and Convalescent Home, Rhyl, upon the fact that Mr. and Mrs. G. W. Hayes, of Worplesdon Hill, Woking, had given £1,000 to found and endow a cot in perpetuity in memory of their son, aged nineteen, who was killed in France, the Bishop of St. Asaph said that while he should be sorry to seem to dictate to anybody as to what form the memorial they might desire to erect to those who had fallen in the war should take, he did venture to say that it would be hard to imagine any memorial more consonant with the suffering that was endured in the war than the establishment of a cot at a children's hospital. It was announced that a free cot had also been founded, called the "Santa Fina" cot, to be maintained by annual subscriptions, for the benefit of the poor children of Manchester. Further accommodation for nurses is urgently required, and when the war is over it is intended to add a wing to the Edith Vizard Nurses' Home, for which funds are already provided. During the year the institution suffered a loss in the death of Mr. Joshua Davies, the honorary treasurer. The vacant office has been filled by Mr. R. M. Hugh Jones, who will combine the financial duties with those of the secretariat.

### AN OBJECTIONABLE EXPEDIENT.

As soon as the supporters of a hospital are taught to regard an urgent need as an actual defect they will generally come forward to remove it. We think it is certainly time that a movement should be made at Liverpool, where the condition of the work at the Hospital for Cancer and Skin Diseases in the absence of a lift is open to serious objection. With a depleted staff and a partially closed out-patient department, with numerous soldiers applying for treatment on their own account, the work under proper conditions would be difficult to cope with. As it is without even a lift, patients have to be carried by nurses from, and perhaps to, the operating theatre or the room where the light apparatus is installed. That is bad for patients, and bad for nurses. An alternative, which is equally open to objection from another point of view, is for the surgeon when the operation is over to carry the patient into the ward. The anæsthetist is also called in as a bearer. Such a state of things, utterly out of keeping with the modern hospital standard, would be sharply criticised at any other time. Nurses, surgeons, and anæsthetists should not have their energies strained and their work interfered with by acting as a substitute for a lift, to say nothing of the unavoidable risk of an accident and of possible injury to the patients. A new lift is estimated to cost £800—how many patients and nurses are to be exposed to accidents until this relatively small sum is raised?

### AN EXPEDIENT TO INCREASE THE NUMBER OF BEDS.

BUILDING schemes, postponed by the war, continue to accumulate. At the Sunderland Eye Infirmary, for instance, owing to an attempt to increase the accommodation for patients, some of the nurses have had to sleep out in rooms found for them. This expedient has been now improved upon by the committee, which has taken several rooms in a building adjoining the hospital, where we may assume that most, if not all, the transferred nurses are now housed. Meantime the erection of a new hospital is of course delayed, but when it can be completed the accommodation will be raised from thirty-six to eighty beds. To meet the estimated cost, which is £20,000, the committee is trying to increase its reserve funds, which at present cannot be an easy matter. But the result of last year's battle between income and expenditure has allowed a balance of £1,893 to be invested for the benefit of the building fund. We are glad to see that workmen's subscriptions have risen by £800 during the past year, and if this progress is maintained the committee should not have long to wait when the moment comes to put their plans into action.

### MISUNDERSTANDINGS AT MERTHYR.

THE deadlock between the Merthyr miners, Dowlais steel-workers, and the committee of the Merthyr General Hospital was discussed at a recent meeting of the Merthyr Trades and Labour Council, under the presidency of Mr. Sam Jennings. The dispute, on which THE HOSPITAL has already offered suggestions, was described to the meeting by Mr. S. Williams, chairman of the Trades Council, and a member of the hospital's board of management. The fight of the workers had been going on for twelve years, but he was afraid they took very little real interest in it. The hospital endowment brought in about £2,000 per annum. At present they wanted about £4,000 per annum. The workers were prepared to contribute 1d. per week on certain conditions. A grievance was that the institution had been monopolised by a few surgeons in the town. It was slightly better now, but they contended that all doctors on the panel should be admitted. To encourage small bodies to contribute they should reduce the qualifying subscription from £15 to £5. Again, all the twelve men on the medical staff were entitled to seats on the executive. The workers asked that the medical staff should be represented by two of their number. The workmen at present had no control. Now the workmen were asked to contribute four times as much as formerly, so, surely, it would not be too much to ask that their representation on the executive should be increased from six to eighteen members. A sub-committee had considered the demands and had agreed to allowing small bodies to be represented if they contributed £5, but would not allow large bodies of workmen contributors to have increased representation on the governors' board. He did not make exactly an impartial statement, as may be judged from his description of the committee as "stubborn and stupid." This is not the language of diplomacy or statesmanship. It is the sort of "plain speaking" which confuses the issue and obscures the facts. The matter is being postponed, and we need only repeat that, while contributing bodies of workmen are certainly entitled to representation, they must never forget that representation is a privilege accorded by a voluntary hospital, not a power to be purchased by a sum of money. The extent of representation is a matter for adjustment, but adjustment can only be found where this principle, surely simple enough, is understood.

### THE MEDICAL EXAMINATION OF R.A.M.C. OFFICERS.

At an inquest held on February 10 at St. Pancras, it was shown that a medical man who was found dead had been admitted only one month before to the R.A.M.C. and gazetted to the rank of captain. Death was shown to be due to fatty degeneration of the heart and liver. With all respect to the board which passed this man for service, and with every allowance for the exceptional circumstances of the times, it is difficult to avoid the impression that this man's constitutional disabilities should have been discovered, since his diseases were so far advanced as to cause sudden death within a month. It seems as if some standardisation is required, for we hear of a very recent case where a healthy man, at a younger age than that of the subject of the aforesaid inquest, has been looked askance at on offering himself for a commission, merely because of slight hæmorrhoids, and is even still uncertain whether he was or was not passed as medically fit for service. It is in no desire to carp that these remarks are offered, but to indicate a need for better organisation and understanding in one small field of medical war work.

### HOSPITALS AND THE USE OF MEDICINAL GLYCERINE.

The Ministry of Munitions announces that owing to the additional demands for glycerine for war purposes it has become necessary to place further restrictions on the issue of medicinal glycerine, and that supplies in future will be reserved for the manufacture of the preparations of the British Pharmacopœia and for such uses of special importance as may be sanctioned by the Ministry of Munitions. These supplies will, however, be small and must be used with the utmost economy. Applications for permit to obtain supplies should be addressed to the Director of Propellant Supplies, 32 Old Queen Street, Westminster, S.W., and should give the following particulars:—(1) Quantity applied for. (2) Stock of glycerine held. (3) Purpose for which supply is required. (In case of extra British Pharmacopœia preparations formulæ should be given.) (4) Applicant's average consumption of glycerine for above purposes. (5) Name and address of proposed suppliers. The medical profession have been informed of the need for economy in prescribing glycerine, and it is anticipated that the requirements for dispensing will be greatly reduced. The stocks of glycerine in the hands of pharmacists should be sufficient to meet these reduced requirements, and, therefore, no glycerine will be issued for dispensing meantime. The surplus stocks held by pharmacists and all stocks held by retailers who are not in a position to use them for these restricted purposes should be disposed of either to other pharmacists who are short of stock or to wholesale houses for making B.P. preparations. It may be questioned whether many institutions have more of this all-important compound in stock than they will need within a reasonable time; but if any have large stocks it is their clear duty to comply with the terms of the Ministry's memorandum.

### AN HEROIC KITCHEN-BOY.

Among the former members of the staff of King Edward VII.'s Hospital, Cardiff, whose names have been inscribed on the Roll of Honour, is A. Lombardini, once a kitchen-boy at the hospital, whose reputation for pluck was first gained in civil life. Some years ago Lombardini succeeded in saving a woman from drowning, and Major-General H. H. Lee has now recorded that Lombardini was well known for his pluck when he joined the Army and preserved his reputation to the end. He died of his wounds. Whatever the form a memorial to the members of the hospital staff who have fallen in the war may take, the name of A. Lombardini should have an honoured place therein as that of a civilian and a soldier who was a man of courage in and out of uniform.

### PRIVATE CHARITY AND PUBLIC REGISTRATION.

The Brighton Bureau for the Mutual Registration of Assistance has had a certain success in obtaining a grant of £50 from the Prince of Wales' National Relief Fund, together with grants both from the Brighton Corporation and the Brighton Board of Guardians. Indeed, it has sufficiently wormed its way into the regard of the local authorities for the suggestion to be made that it should be established on a permanent basis, which appears to mean under semi-official or municipal control. But the bureau is still regarded with apathy by many benevolent persons, who prefer to do good by stealth and, in effect, decline to register the cases which they help. We are glad to see that Mr. H. L. Woollcombe, of the C.O.S., pointed out the evil of overvisiting, which he rightly declared to be as objectionable as overlapping in relief, and, of course, is a much greater nuisance to the poor.

### WHO SHOULD BE FIRST?

If one glances at a dozen annual reports of a dozen different hospitals one will find that after the names of the patrons come the names of the president and the vice-presidents. So far, everything is in order, but there seems to be a difference of opinion as to whether the name of the chairman should be printed above the name of the treasurer, or *vice versâ*. When one individual fills both offices there is, of course, no difficulty. In some instances, where the treasurer happens to be a man of title and the chairman is not, there is an excuse for putting the treasurer's name before that of the chairman. Few hospital secretaries will urge that the work of a treasurer is anything like so continuous or important as that of the chairman. This, then, is the answer to the point. For if a chairman does more work for a hospital than a treasurer, he is entitled to the first place. Yet there are occasions when a treasurer gets the pull of the chairman. The treasurer's name probably appears much more frequently in public announcements than does the chairman's. It is true that the chairman makes the appeal in most cases, just as it is equally true that the treasurer, on behalf of his colleagues, makes the acknowledgments. And everybody will agree that acknowledgments are more numerous than appeals. In nine cases out of ten a chairman is looked to for much more work of a creative character than is a treasurer, though it must be admitted that the latter honorary official has a larger share of legal matters claiming his attention, especially when the institution is one that receives a large amount yearly in legacies. To sum up, the chairman would seem to win this friendly little bout on points.

### SAUSAGES UNDER SURVEILLANCE.

Dr. A. W. J. MacFadden's report on the work of the Inspectors of Foods during the year 1915-1916 states that special attention has been given to firms engaged in the preparation of meat foods, such as brawn, sausages, meat-pies, and potted meats, on account of the greater liability of these articles of food, if prepared under insanitary conditions, to endanger the health of the consumer. These firms may be roughly divided into two classes, firstly, the large firms who specialise in the preparation of articles of this class, and, secondly, the butcher or baker who makes sausages, brawn, or pies in connection with his business. The premises of the first class are as a rule found to be fairly good, or if not quite satisfactory, can without much trouble be made so. The second class, however, are found to present some remarkable contrasts. In many of them the cleanliness of the premises and the methods employed leave nothing to be desired; in others the conditions can only be described as deplorable. In one case of this kind the sausage-preparing room was a cellar apartment with no ventilation at all, the walls being damp and mouldy, the floor having patches of loose rubble, and the table cracked and fouled by mice and other dirt. Cases of this sort have been at once reported to the Army Canteen Committee, with the result that supplies to troops have been stopped forthwith. Other cases have been discovered where the sausage machine has been installed in a slaughterhouse, and wherever this practice has been found the proprietor has been warned to make other arrangements immediately if he wished to continue to supply food to troops. As a rule, after a visit by an Inspector of Foods firms engaged in making these kinds of foods endeavour to comply with any suggestions made to them for the improvement of their premises, and on a second visit being made a very marked improvement has generally been noted.

### WELFARE WORK IN MONMOUTHSHIRE.

A lady's fine gift in the interests of maternal and child welfare was reported by Lady Mather-Jackson to the annual meeting, held lately, of the Monmouthshire Nursing Association. The progress made by the Association recently is due in no small degree to the energy and organising ability shown by Lady Mather-Jackson, and it was only fitting that grateful reference should be made to what she has been able to accomplish. A great extension of the work has taken place. In some of the populous parts of the country district branches have been opened, and ere long twenty infant welfare centres will be established. The announcement of an annual subscription of £200 from Mrs. Hanbury, of Pontypool Park, towards the maintenance of nurses at Pontypool and Panteg was naturally received with enthusiasm by the meeting.

### FOOD REGULATIONS FOR INSTITUTIONAL STAFFS.

The West Bromwich Board of Guardians has instructed the medical superintendent and the master to adopt the Food Controller's suggestions in regard to the consumption of meat, bread, and sugar, and that such substitutes be chosen as will, so far as possible, help to reduce the number of imported foods. The medical superintendent has, of course, been given a free hand in regard to the patients. The plan adopted in regard to the staff translates the somewhat vague propositions mentioned above into an effort to limit the consumption of meat, bread, and sugar to a fixed amount per week. This total is arrived at by starting with the amounts suggested by the Food Controller as sufficient for any individual, and then multiplying it by the number of the staff. It would be interesting to know what substitutes are adopted, and how far such substitutes themselves are home-grown.

### WHAT SHALL WE EAT?

The thoughts of institutional managers are this week chiefly turned towards food. Close on the announcement of the meatless day at the British Home and Hospital at Streatham comes the news that the sister institution at Putney has notified the patients and staff by poster that the Food Controller's instructions will be rigorously carried out, and that the provisions purchased will not exceed the amount specified. The small allowance of meat is everywhere agreed to be the chief difficulty, and little comfort is obtained by surveying the markets for substitutes for this form of food. Fish is scarce and dear. The *Fish Trades Gazette* gives a list of current prices as compared with three years ago. Cod has increased from an average of 3s. 9d. to 9s. 9d. per stone, fresh herrings from 18s. 9d. to 70s. per barrel, and even the homely shrimp has gone up 5s. per bushel. The only thing that has gone down in this market is oysters. Now we are faced by a deadlock in the potato trade; a margin of £6 per ton between the farmer and the retailer is considered all too small for profit of the middleman and freight charges, and the Food Controller must be realising the difficulty and danger of fixing maximum prices. One result of all these troubles is that the work and anxiety of the caterers of our institutions is doubled.

### MUNICIPALITIES MAY PAY THE DOCTOR.

A REPORT presented to the Barnes Council by the Health Committee makes it clear that in certain circumstances municipalities can pay the doctor's bill. The committee comes to report on the matter because it proposes a welfare scheme, and, for the information of the members of the Council, the powers of local authorities under the Memorandum by the Medical Officer of the Local Government Board of November 1915 are:—The local authority may provide beds for patients suffering from complications arising during pregnancy or childbirth. These beds can be provided directly by the local authority or by arrangement between them and an existing hospital. The necessity of reserving hospital beds for the treatment of puerperal fever and allied conditions must be borne in mind. A local authority is also empowered, with the Board's consent, to pay in whole or in part the fee for the doctor or midwife attending the confinement at home of necessitous women, and one half of such approved expenditure is paid by the Local Government Board.

### MORE SUPERVISION OF MIDWIVES.

THE Local Government Board has recently investigated the arrangements for the inspection of midwifery in London, and has made representations to the effect that, whilst the two inspectors of midwives carry out their duties with great zeal and ability, they are unable, in the time at their disposal, to perform adequately all the duties necessary in connection with midwives in London, and the Board has invited the County Council to consider the question of substantially increasing the staff available for the purpose at the earliest favourable opportunity. Reporting upon this, the Midwives Act Committee mentions that during the past two years the growth of the work which ought to be performed has been very great, and it has been found to be quite impossible in face of the increase of work to investigate fully all cases of puerperal fever, ophthalmia neonatorum, etc.; nor have the inspectors been able to make the necessary routine visits to midwives and to inspect their practices. Having had particulars of the amount of work carried out in 1915 and 1916, the committee remarks that it appears that the number of visits which should be paid during a year is more than double the number of visits made by the inspectors in each of these two years, notwithstanding the fact that a considerable amount of overtime was worked by the inspectors. Moreover, if certain suggestions made by the Central Midwives Board with regard to the visitation of cases of stillbirth be adopted, the work will be yet further increased. The committee realises that the services of persons possessing medical qualification are at the present time required by the State in other spheres of labour, but feels that the Council will be justified in taking into its service additional women doctors for such important work affecting maternal and infant life.

### CLAIMS FOR REBATE OF SPIRIT DUTY.

IT is not generally understood by the hospitals that applications will now be considered for the rebate of duty on medicinal alcohol used during 1916; indeed, up to a few days ago the Local Government Board had received only two requests for the forms on which the return must be made. This apparent slackness on the part of hospital secretaries and pharmacists is not caused by lack of keenness to obtain the rebate, but must rather be attributed to the fact that no public announcement has been made that the authorities are ready to receive applications, which should be made to the Secretary, Local Government Board, Whitehall, S.W.

### THIS WEEK'S DRUG MARKET.

THE week has been an interesting one in the drug market, and a number of price changes have taken place. Makers of bromides have reduced their quotations materially; the market had not been firm for some time past, and it is mainly as a result of increasing competition from foreign sources that English makers have made this concession. The price of English refined camphor has been advanced, but even the higher prices are below those that are being paid for Japanese refined. As a consequence of the rise in the value of citric acid slightly higher figures are now quoted for potassium citrate, sodium citrate, and iron and ammonium citrate. Tartaric acid is also dearer. Much higher prices are asked for quicksilver, and it is difficult to do business in this metal; as a consequence the value of calomel, corrosive sublimate, and other salts of mercury will no doubt be raised. The activity in quinine has almost subsided, and prices are rather lower again. A large business has been done in phenacetin, and the price tendency is still in a downward direction. Acetylsalicylic acid is also tending downwards in price, but no substantial decline is expected at the moment. Sarsaparilla is dearer for all varieties. Myrrh is very scarce, especially the better qualities. The price of almond oil has been advanced. No news is to hand concerning the Norwegian cod-fishing, and one rarely hears cod-liver oil mentioned on the drug market, the price being almost prohibitive.

